# Postexamination item analysis of undergraduate pediatric multiple-choice questions exam: implications for developing a validated question Bank

**DOI:** 10.1186/s12909-024-05153-3

**Published:** 2024-02-21

**Authors:** Nagwan I. Rashwan, Soha R. Aref, Omnia A. Nayel, Mennatallah H. Rizk

**Affiliations:** 1 Pediatrics, Qena Faculty of Medicine, South Valley University, Qena, Egypt; 2 Community Medicine, Faculty of Medicine, Alexandria University, Alexandria, Egypt; 3 Clinical Pharmacology, Faculty of Medicine, Alexandria University, Alexandria, Egypt; 4 Medical Education, Faculty of Medicine, Alexandria University, Alexandria, Egypt

**Keywords:** Single best answer questions, Extended matching questions, Item analysis, Item writing flaws, Question Bank

## Abstract

**Introduction:**

Item analysis (IA) is widely used to assess the quality of multiple-choice questions (MCQs). The objective of this study was to perform a comprehensive quantitative and qualitative item analysis of two types of MCQs: single best answer (SBA) and extended matching questions (EMQs) currently in use in the Final Pediatrics undergraduate exam.

**Methodology:**

A descriptive cross-sectional study was conducted. We analyzed 42 SBA and 4 EMQ administered to 247 fifth-year medical students. The exam was held at the Pediatrics Department, Qena Faculty of Medicine, Egypt, in the 2020–2021 academic year. Quantitative item analysis included item difficulty (P), discrimination (D), distractor efficiency (DE), and test reliability. Qualitative item analysis included evaluation of the levels of cognitive skills and conformity of test items with item writing guidelines.

**Results:**

The mean score was 55.04 ± 9.8 out of 81. Approximately 76.2% of SBA items assessed low cognitive skills, and 75% of EMQ items assessed higher-order cognitive skills. The proportions of items with an acceptable range of difficulty (0.3–0.7) on the SBA and EMQ were 23.80 and 16.67%, respectively. The proportions of SBA and EMQ with acceptable ranges of discrimination (> 0.2) were 83.3 and 75%, respectively. The reliability coefficient (KR20) of the test was 0.84.

**Conclusion:**

Our study will help medical teachers identify the quality of SBA and EMQ, which should be included to develop a validated question bank, as well as questions that need revision and remediation for subsequent use.

**Supplementary Information:**

The online version contains supplementary material available at 10.1186/s12909-024-05153-3.

## Introduction

“Assessment affects students learning in at least four ways: its content, format, timing, and any subsequent feedback given to the medical students” [1]. MCQs are a well-established format for undergraduate medical student assessment, given that MCQs allow broad coverage of learning objectives. In addition, MCQs are objective and scored easily and quickly with minimal human-related errors or bias. Well-designed MCQs allow for the assessment of higher cognitive skills rather than low cognitive skills [2].

However, MCQs have some limitations. Construction of MCQs is most difficult and time-consuming even for well-trained staff members. There is evidence that the basic item-writing principles are not followed mostly when constructing MCQs. The presence of flawed MCQs can interfere with the accurate and meaningful interpretation of test scores and negatively affect student pass rates. Therefore, to develop reliable and valid tests, items must be constructed that are free of such flaws [3].

Item analysis (IA) is the set of qualitative and quantitative procedures used to evaluate the characteristics of items of the test before and after test development and construction. Quantitative item analysis uses statistical methods to help make judgments about which items need to be kept, reviewed, or discarded. Qualitative item analysis depends on the judgment of the reviewers about whether guidelines for item writing are followed or not [4].

In quantitative IA, three psychometric domains are assessed for each item: item difficulty (P), item discrimination (D), and distractor efficiency (DE) [5]. Item difficulty (P) refers to the proportion of students who correctly answered the item. It ranges from (0–1) [6]. Item discrimination (D) indicates the extent to which the item can differentiate between higher- and lower-achieving students. It ranges between − 1.0 (perfect negative discrimination) to + 1.0 (perfect positive discrimination) [6]. An item discrimination of more than 0.2 was reported as evidence of item validity. Any item with less than 0.2 or negative discrimination should be reviewed or discarded [7, 8]. Distractor efficiency (DE) is determined for each item based on the number of nonfunctioning distractors (NFDs) (option selected by < 5% of students) within it [9].

Qualitative IA should be routinely performed before and after the exam to review test items’ conformity with MCQ construction guidelines. The two most common threats to the quality of multiple-choice questions are item writing flaws (IWFs) and testing of lower cognitive function [10]. Item writing flaws are violations of MCQ construction guidelines meant to prevent testwiseness and irrelevant difficulty from influencing medical students’ performance on multiple-choice exams. IWFs can either introduce unnecessary difficulty unrelated to the intended learning outcomes or provide cues that enable testwise students to guess the correct answer without necessarily understanding the content. Both types of flaws can skew the final test scores and compromise the validity of the assessment [8, 11]. Well-constructed MCQs allow the evaluation of high-order cognitive skills such as the application of knowledge, interpretation, or synthesis rather than testing lower cognitive skills. On the other hand, MCQs were mostly used to test lower rather than higher cognitive skills, which can be considered a significant threat to the quality of multiple-choice questions [12]. In many medical schools, faculty members are not sufficiently trained to construct MCQs that examine high cognitive skills linked to authentic professional situations [13].

This study aimed to perform a postexamination quantitative and qualitative item analysis of two types of MCQs, SBA and EMQ, to provide guidance when making decisions regarding keeping, reviewing, or discarding questions from exams or question banks.

## Methods

### Participants

Data were collected from the pediatric summative exam of Pediatrics course (PED502, a 7-credit-hour course), which was conducted at the Qena Faculty of Medicine, South Valley University, Qena, Egypt. The medical school implements a ‘6 + 1’ medical curriculum. This is a comprehensive seven-year educational program that includes 6 years of foundational and clinical medical education, followed by a year of practical training or internship. Qena Faculty of Medicine, South Valley University has been officially accredited by the National Authority for Quality Assurance and Accreditation of Education (NAQAAE) in 2021 (https://naqaae.eg/ar/accredited_organization/accredited_he). Approximately 247 medical students in their fifth year were qualified to take the pediatric final exam during the second semester of the 2020–2021 academic year. All exam questions were authored by Pediatrics department, Qena Faculty of Medicine, South Valley University faculty members, intended to have one correct response.

### Procedures

The exam papers and relevant SBA and EMQ item analysis reports were collected and reviewed. Outputs of Remark Classic OMR® (MCQ test item analysis software) were used for scanning and analyzing data from the exam. It automates the process of collecting and analyzing data from “fill in the bubble” forms. The information collected were the following: test item analysis report; number of questions graded, students’ responses (correct, incorrect, no response), item difficulty (P), item discrimination (D), and distractor efficiency (DE). The qualitative item analysis was determined by three assessors. They were provided with MCQ qualitative analysis checklist to review the exam (Additional file 1). Two types of multiple-choice questions (MCQ) were used in this exam; Single Best Answer (SBA) and Extended Matching Questions (EMQs). SBA items were 42 with five options, and the EMQs were four sets with three stems in each set and eight options for each set. The correct response was awarded one and half mark and the incorrect response given zero mark. Each SBA and EMQ were analyzed independently by three assessors as to its level of cognitive skill test and presence of item writing flaws. Assessors had content-area expertise, experience preparing multiple choice exam. Questions were categorized according to modified Bloom’s taxonomy: Level I Knowledge (recall of information), Level II Comprehension and Application (ability to interpret data). Level III Problem solving (Use of knowledge and understanding in new circumstances) [14]. Cohen’s κ was run to determine the inter-rater reliability for the three assessors which was found to be substantial, with a Kappa coefficient of 0.591 (*p* <  0.001). This indicates that there is a significant level of agreement between the assessors beyond what would be expected by chance according to the guidelines proposed by Landis and Koch (1977) [15].

 SBA item writing flaws (IWFs) were retrieved from NBME item writing guide (6th edition, 2020) [11]. IWFs were categorized and scored as stem flaws (1 = negatively phrased stem, 2 = logical/grammatical cue, 3 = vague, unclear term, 4 = tricky, unnecessarily complicated stems, 5 = no led in question/defective, 6 = poorly constructed, short). Option Flaws (1 = Long, complex options, 2 = Inconsistent use of numeric data, 3= “None of the above” option, 4 = Nonhomogeneous options, 5 = Collectively exhaustive options, 6 = Absolute terms, 7 = Grammatical/logical clues, 8 = Correct answer stands out, 9 = Word repeats (clang clue), 10 = Convergence). EMQ IWFs were retrieved from Case and Swanson (1993) work that highlighted the characteristics of well written EMQs [16]. EMQ IWFs were categorized and scored into: Options Flaws (1 = options less than 6/more than 25, 2 = not focused, 3 = no logical/alphabetical order, 4 = not homogenous, 5 = overlapping/complex), Led in Question Flaws (1 = not clear/focused, 2 = nonspecific), and Stem Flaws (1 = non-vignette, 2 = not Clear/Focused vignette, 3 = short, poorly constructed).

### Data analysis

Descriptive methods are based on Classical Test Theory (CTT). The CTT considers reliability, difficulty, discrimination, and the distractor efficiency to check the appropriateness and plausibility of all distractors. The core of this theory is based on the functions of the true test score and the error of random measurement [17]. Item psychometric parameters were collected from reported examination statistics including item difficulty (P), item discrimination (D), distractor efficiency (DE) and internal consistency reliability for the whole test. The criteria for classification of item difficulty are as follows: *P* <  0.3 (too difficult), P between 0.3 and 0.7 (good/acceptable/average), *P* > 0.7 (too easy) and item difficulty between 0.5 and 0.6 (excellent/ideal). The criteria for classification of the item discrimination are as follows: D ≤ 0.20 (poor), 0.21 to 0.39 (good) and D ≥ 0.4 (excellent). The items were categorized on the basis of numbers of NFDs in SBA and EMQ, that is, if a five-option SBA includes 4-NFD, 3-NFD, 2-NFD, 1-NFD, or 0-NFD, the corresponding distractor efficiency (DE) is 0.00, 25, 50, 75 and 100%, respectively. In an EMQ, if the options include 7-NFD, 6-NFD, 5-NFD, 4-NFD, 3-NFD, 2-NFD, 1-NFD, or 0-NFD, the corresponding distractor efficiency (DE) is 0.00, 14.30, 28.50, 42.80, 57.10, 71.40, 85.70, and 100.00%, respectively.

### Test reliability

Reliability refers to how consistent the results from the test are. The Kuder and Richardson method KR-20 is a measure of reliability for a test with binary variables (i.e. answers that are right or wrong). K-R20 is used to estimate the extent to which performance on an item relates to the overall test scores. In this study, K-R20 was used to estimate the reliability of the pediatric final exam. A single test was used hence the reliability method rest in the internal consistency methods*.* The scores for KR-20 range from 0 to 1, where 0 is no reliability and 1 is perfect reliability. The value of KR-20 between 0.7 and 0.9 falls in good range. Reliability estimates can be applied in numerous ways in assessment. A practical application of the reliability coefficient is to compute the Standard Error of Measurement (SEM). The SEM is calculated for the full range of scores on an evaluation using a specific formula, *SEM* = *Standard deviation* ×  √ (1 − *Reliability*). This SEM can be utilized to create confidence intervals around the observed assessment score, which signifies the accuracy of the measurement, considering the reliability of the evaluation, for each scoring level. This estimate aids assessors in determining how an individual’s observed test score and true score differ [18].

Basic frequency distributions and descriptive statistics were computed for all variables. Normality assumption testing involved the use of Q-Q plots, frequency histograms (with normal curve overlaid) and Shapiro-Wilks Test of Normality. This testing found that Normality was met for all analyses except one variable (difficulty level of SBA). This variable was subjected to a two-step normalization process to achieve a normal distribution, as per the method outlined by Templeton, Gary F. (2011). This approach ensured a more accurate analysis of the data [19].

Parametric significance test, specifically the independent t-test, was used to compare the means of difficulty and discrimination, for SBA and EMQ formats. The independent t-test allowed us to determine if there were statistically significant differences in difficulty and discrimination between the SBA and EMQ formats. All analyses were conducted as two-tailed, with *p* = .05 used as the threshold for statistical significance, using SPSS Statistics for Windows, Version 24 (IBM Corp.). Figure was generated in Microsoft Excel 2013.

## Results

The final pediatrics exam was composed of 54 items, and the total score was 81 (1.5 mark for each question). The mean exam score was 55.04 ± 9.82. The value of KR-20 was 0.86. This is considered acceptable as it is greater than the commonly accepted threshold of 0.7 for acceptable reliability. This suggests that the MCQ exam in this study is a reliable tool for assessment [20]. Reliability depends both on Standard Error of Measurement (SEM) and on the ability range (standard deviation, SD) of students taking an assessment. The standard error of measurement (SEM) was 3.91. The smaller the SEM, the more accurate are the assessments that are being made [21].

### Quantitative item analysis

The difficulty level of items was easy (*P* > 0.7), at 61.9% of SBA and 66.69% of EMQ. The difficulty level of items was moderate (0.7 ≥ *P* > 0.3) at 23.8% of SBA and 16.67% of EMQ. However, the difficulty level was difficult (*P* ≤ 0.3), at 14.3% for SBA and 16.67% for EMQ. Item discrimination was > 0.2 at 83.3% of SBA and 75% of EMQ, indicating good discriminating items. Three SBAs (7.10%) had poor discrimination (D ≤ 0.2). Four SBAs (9.5%) had negative discrimination. The mean DE of SBA was 37.69% ± 33.12. The percentage of functioning distractors was 36.9%, and the percentage of nonfunctioning distractors was 63.1%. Only 11.90% of SBA had distractor efficiency (100.00%), while 26.20% had distractor efficiency 0.00%. The mean DE for EMQ was 13.09 ± 15.46. No EMQ had a DE of 100%, while EMQ with DE (0.00%) was 41.60%. The percentage of functioning distractors was 13.1%, while the percentage of nonfunctioning distractors was 86.90%.

Table 1 indicates that only 9 SBAs (21.4%) met the recommended levels for difficulty and discrimination (with P ranging from 0.3 to 0.7 and D > 0.2). Table 2 indicates that only 2 EMQ items (16.7%) met the recommended levels for difficulty and discrimination (with P ranging from 0.3 to 0.7 and D > 0.2). These questions should be retained in the question bank, provided they are free of IWFs.

**Table 1:** Distribution of single best answer (SBA) items by difficulty and discrimination levels: frequencies and percentages (42 SBAs)

IA parameter			Item Difficulty		
			0.71–1	0.3–0.7	< 0.3
			Easy	Moderate	Difficult
Item Discrimination	≥ 0.4	Very good	12 (28.6%)	4 (9.5%)	0
	0.21 to 0.39	Good	13 (30.9%)	5 (11.9%)	1 (2.4%)
	0–0.2	Poor	1 (2.4%)	1 (2.4%)	1 (2.4%)
	Negative	Negative	0	0	4 (9.5%)

**Table 2:** Distribution of extended matching question (EMQ) items by difficulty and discrimination levels: frequencies and percentages (12 EMQ stems)

IA parameter			Item Difficulty		
			0.71–1	0.3–0.7	< 0.3
			Easy	Moderate	Difficult
Item Discrimination	≥ 0.4	Very good	6 (50%)	0	0
	0.21 to 0.39	Good	1 (8.3%)	2 (16.7%)	0
	0–0.2	Poor	1 (8.3%)	0	2 (16.7%)
	Negative	Negative	0	0	0

Table 3 shows the comparative analysis the mean difficulty (P) and discrimination (D) values of Single Best Answer (SBA) and Extended Matching Questions (EMQ), the following findings were observed. The mean difficulty for SBA was 0.67 (±0.28) and for EMQ was 0.70 (±0.28). The independent t-test showed no significant difference between the two formats (t = − 0.405, *p* = 0.686). Similarly, the mean discrimination for SBA was 0.32 (±0.16) and for EMQ was 0.35 (±0.18). Again, the independent t-test revealed no significant difference (t = − 0.557, *p* = 0.620).

**Table 3:** Comparison of the mean difficulty P and discrimination D values of the SBA and EMQ

IA parameter	SBA Mean ± SD	EMQ Mean ± SD	t-test	*P*-Value
P	0. 67 ± 0.28	0.70 ± 0.28	−0.405^t^	0.686*
D	0.32 ± 0.16	0.35 ± 0.18	-0.557^t^	0.620*

*SD* Standard deviation t: independent t-test *: not statistically significant (*p* > 0.05)

### Qualitative item analysis

The prevalence of SBA testing low cognitive skills was 76.19%. Only 23.8% of SBAs tested higher cognitive skills. Conversely, most EMQs tested higher cognitive skills (75%), and 25% of EMQs tested low cognitive skills.

The frequency of flawed SBAs with stem flaws was 30 questions (71.40%). Option flaws were found in 23 questions (54.76%). SBA with more than 2 IWFs comprised 15 questions (35.7%). Poorly constructed stems were the most frequent stem flaw, with 15 questions (35.70%), followed by negatively phrased stems (33.30%), vague, unclear terms (21.40%), tricky unnecessarily complicated stems (21.40%), no lead-in question (21.40%) and logical/grammatical cue flaws (7.10%). Regarding the flaws related to options**,** the nonhomogeneous options list was the most frequent flaw (35.70%). The correct answer stands out, with long complex options and inconsistent use of numeric data (9.50%) each. Word repeats and convergence were found in 4.80% of cases each.

EMQs with option flaws with no logical/alphabetical order were the most frequent (100.00%), nonhomogenous, and overlapping/complex (25.00%) for each. Lead-in statement flaws included unclear/unfocused lead-in statements (75.00%) and nonspecific statements (50.00%). The stem flaws found were nonvignette and short poorly constructed stems (25.00%) for each.

**Fig. 1:**
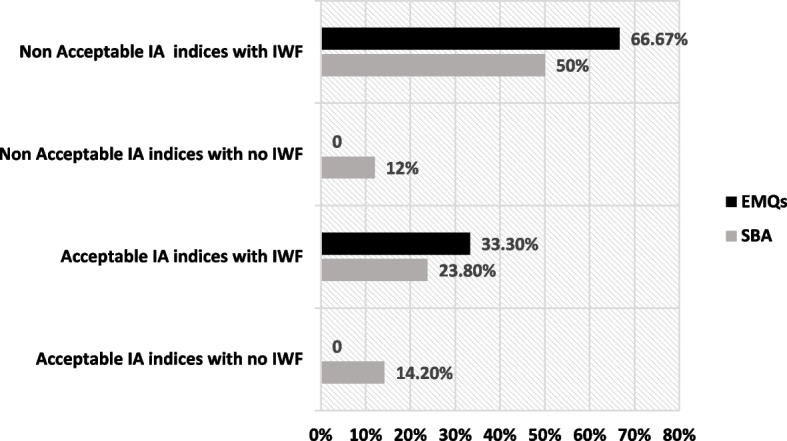
The four categories of both SBA and EMQ formats based on: the level of item analysis (IA) indices (difficulty P and discrimination D) and the presence or absence of IWFs

Figure 1 shows the four categories of MCQ based on the level of IA indices (P and D) and the presence or absence of IWFs. The four categories are as follows:

I. Acceptable IA indices with no IWFs: Questions have difficulty level within the acceptable range (0.3–0.7) and discrimination level > 0.2, and items are free of flaws.
II. Acceptable IA indices with IWFs: Questions have acceptable difficulty and discrimination levels, and items are flawed.
III. Nonacceptable IA indices with no IWFs: Questions have difficulty level < 0.3 or more than > 0.7 and discrimination less than < 0.2, and items are free of flaws.
IV. Nonacceptable IA indices with IWFs: Questions have difficulty level < 0.3 or more than > 0.7 and discrimination less than < 0.2, and items are flawed.

The prevalence of SBA and EMQ with acceptable IA indices with no IWFs was 14.2 and 0%, respectively. Those previous questions should be kept in the questions bank without any modifications. However, the prevalence of SBA and EMQ with acceptable IA indices with IWFs was 23.8 and 33.3%, respectively. These questions need remediations before being kept in the question bank. Items with nonacceptable IA indices with or without IWFs (which constitute more than 60% of the items) should be discarded from the question bank.

## Discussion

In this study, we performed both quantitative and qualitative postexamination item analysis of the summative undergraduate pediatrics MCQ exam. The quantitative analysis discovered a range of item difficulty and discrimination levels, highlighting the importance of a diverse question bank in assessing a broad spectrum of student abilities. Qualitative item analysis, on the other hand, involves a more subjective review of each item. It helped to identify issues with cognitive level, item clarity, and writing flaws. The qualitative analysis complemented the quantitative findings and provided additional insights into the quality of the items. The findings underlined the value of both quantitative and qualitative item analysis in ensuring the validity and reliability of the exam and in building a robust question bank. Both quantitative and qualitative item analysis are crucial for making decisions about whether to keep, review, or remove questions from the test or question bank. These decisions enabled us to identify ideal questions and develop a valid and reliable question bank for future assessment that will enhance the quality of the assessment in undergraduate pediatrics.

An ideal MCQ is clear, focused, and relevant to the intended learning outcomes. It should have a single best answer and distractors that are plausible but incorrect. In addition, an ideal MCQ should have an appropriate level of difficulty and discrimination power. The findings from this study suggest that the proportion of ideal questions, as defined by the three criteria (difficulty level of 0.3–0.7, discrimination level > 0.2, and 100% distractor efficiency), is lower than what has been reported in previous studies. Specifically, only 4.7% of Single Best Answer (SBA) questions met these criteria, and none of the Extended Matching Questions (EMQs) did. This is in contrast to previous studies, which reported that 15–20% of MCQs fulfilled all three criteria [22, 23]. These findings highlight the importance of rigorous question development and review processes to ensure the quality of MCQs. This could include strategies such as regular postexamination item analysis, peer review of questions, and ongoing training for question authors [24, 25].

In this study, the mean P was higher for the EMQ than for the SBA, although the difference was not statistically significant (t = − 0.405, *p* = 0.686). The mean D was higher for the EMQ than for the SBA, although the difference was not statistically significant difference (t = − 0.557, *p* = 0.620). Therefore, both formats demonstrated comparable levels of difficulty and discrimination in the context of this study. This is in contrast to previous studies, which have reported significant differences in difficulty levels between these two formats. Increasing the number of options had an influence on difficulty levels as questions with more options were more difficult or harder [26, 27]. This discrepancies could be explained by high number of non-functioning distractors (NFD) in Extended Matching Questions (EMQ) which had a significant impact on both the item difficulty and discrimination levels of the questions. Firstly, the presence of NFDs leads to easier questions. Therefore, a high number of NFDs can make it easier for examinees to identify the correct answer. Secondly, NFDs can also affect the item discrimination level. If a question has many NFDs, it may not effectively discriminate between higher- and lower-achieving students. In this study, the percentage of non-functioning distractors of EMQs was 86.90%. These findings underline the importance of careful distractor selection and review in the development of EMQs. By reducing the number of NFDs, it may be possible to increase the item difficulty and discrimination levels of the questions, thereby improving the overall quality of the assessment.

Distractor analysis of MCQs can enhance the quality of exam items. We can fix MCQ items by replacing or removing nonfunctioning distractors rather than eliminating the whole item, which would save more energy and time for future exams [24]. In both the SBA and the EMQ, we found a considerable number of nonfunctioning distractors (NFDs), 63.10 and 86.90%, respectively. We found that our faculty members need training for the construction of plausible distractors of MCQs to improve the quality of MCQ exams [28]. In addition, we should reduce the number of options to three-option items instead of five-option items [29, 30]. Tarrant and Ware proved that three-option items perform equally well as four-option items and have suggested writing three-option items, as they require less time to be developed [31]. NFDs were more commonly encountered in EMQ than SBA. The EMQ had more options (8 compared to 5), so it may be more difficult to create plausible distractors that draw students to respond to them. All EMQ with many NFDs should be revised or even converted to SBA instead [32].

The reliability coefficient (KR20) of the test was 0.84, which shows an acceptable level of reliability. The standard error of measurement (SEM) was 3.91. SEM estimates the amount of error built into a test taker’s score. This estimate aids evaluators in determining how an individual’s observed test score and true score differ. The test reliability and the SEM are interconnected. The SEM decreases as the test reliability increases [5]. For a short test (fewer than 50 items), a KR20 of 0.7 is acceptable, while for a prolonged test (more than 50 items), a KR20 of 0.8 would be acceptable. Test reliability can be improved by the removal of flawed items or very easy or difficult items. Items with poor correlation should be revised or discarded from the test [7].

In our study, we analyzed the cognitive levels of SBA and EMQ based on modified Bloom’s taxonomy [14]. We found that 76.19% of SBA assessed low cognitive levels, while only 25% of EMQ assessed low cognitive skills. Conversely, 75% of EMQ assessed higher cognitive skills. These results are similar to other studies that found that 60.47 and 90% of MCQs were at low cognitive levels [13]. EMQs are recommended to be used in undergraduate medical examinations to test the higher cognitive skills of advanced medical students or in high-stakes examinations [33]. A mixed examination format including SBA and EMQ was the best examination to distinguish poor from moderate and excellent students [34].

In this study, we aimed to find common technical flaws in the MCQ Pediatrics exam. We found that only 26.20% of SBA questions followed all best practices of item writing construction guidelines. The prevalence of item writing flaws was 73.80% for SBA, and all EMQ sets were flawed. This high proportion of flawed items was similar to other studies, where approximately half of the analyzed items were considered flawed items [35]. The high prevalence of IWFs in our study exposed the lack of preparation and time devoted by evaluators for MCQ construction. The most prevalent types of flaws in SBA questions were poorly constructed, short stems (35.70%), and negatively phrased stems (33.3%). Furthermore, all EMQ had flaws, and option flaws were the dominating type of flaws (100.00% no logical order, 25.00% nonhomogeneous, and 25.00% complex option). These findings were consistent with other studies [13, 35].

The presence of IWFs had a negative effect on the performance of high-achieving students, giving an advantage to borderline students who probably relied on testwiseness [36]. According to Downing, MCQ tests are threatened by two factors: construct-irrelevant variance (CIV) and construct underrepresentation (CUR). Construct-irrelevant variance (CIV) is the incorrect inflation or deflation of assessment scores caused by certain types of uncontrolled or systematic measurement error. Construct underrepresentation (CUR), which is the cognitive domain’s down sampling. Flawed MCQs tend to be ambiguous, unjustifiably difficult, or easy. This is directly related to the CIV added to a test due to flawed MCQs. CUR takes place when many of the test items are written to assess low levels of the cognitive domain, such as recall of facts [37]. All defective items found by quantitative item analysis should be analyzed for the presence of item writing flaws. Those defective items need to be correctly reconstructed; validated and feedback should be given to the item’s authors for corrective action. Both quantitative and qualitative item analysis are necessary for the validation of viable question banks in undergraduate medical education programs [38].

## Limitations and delimitations

### Limitations

1. Subjectivity in Qualitative Analysis: While the qualitative item analysis provided valuable insights, it is inherently subjective. Different assessors might have different interpretations of item clarity, cognitive level, and writing flaws. This subjectivity could potentially impact the consistency of the analysis.
2. Scope of the Study: The study was limited to a single summative undergraduate pediatrics MCQ exam. Therefore, the findings may not be generalizable to other exams or disciplines.
3. Sample Size: The study’s conclusions are based on the analysis of a single exam. A larger sample size, including multiple exams over a longer period, might provide more robust and reliable findings.

### Delimitations

1. Focus on MCQs: The study was delimited to two types of multiple-choice questions (MCQs). Other types of questions, such as short answer or essay questions, were not included in the analysis.
2. Single medical school Study: The study was conducted within a medical school, which may limit the generalizability of the findings to other medical schools with different student populations or assessment practices.

Despite these limitations and delimitations, the study provides valuable insights into the importance of both quantitative and qualitative item analysis in ensuring the validity and reliability of exams and in building a robust question bank. Future research could aim to address these limitations and delimitations to further enhance the quality of MCQ assessment in undergraduate medical education.

## Conclusions

In summary, item analysis is a vital procedure to ascertain the quality of MCQ assessments in undergraduate medical education. We demonstrated that quantitative item analysis can yield valuable data about the psychometric properties of each item. Furthermore, it can assist us in selecting “ideal MCQs” for the question bank. Nevertheless, quantitative item analysis is insufficient by itself. We also require qualitative item analysis to detect and rectify flawed items. We discovered that numerous items had satisfactory indices but were inadequately constructed or had a low cognitive level. Hence, both quantitative and qualitative item analysis can enhance the validity of MCQ assessments by making informed judgments about each item and the assessment as a whole.

### Supplementary Information

**Supplementary Material 1.**

## Data Availability

Primary data are available from the corresponding author upon reasonable request.

## Abbreviations

MCQ: multiple choice question
IA: item analysis
P: item difficulty
D: item discrimination
DE: distractor efficiency
SBA: single best answer
EMQs: extended matching questions
IWFs: item writing flaws.
