# Supplementary material for: Postexamination item analysis of undergraduate pediatric multiple-choice questions exam: implications for developing a validated question Bank
Source: BMC Med Educ. 2024 Feb 21;24:168. doi: 10.1186/s12909-024-05153-3 (PMC10882907; doi:10.1186/s12909-024-05153-3)
Supplement: Supplementary file 1 — Supplementary Material 1. [file 12909_2024_5153_MOESM1_ESM.pdf]

Evaluation of End of Semester Objective Written Exam Paper

I. **General Outlines of the test paper & Instructions for Students**			
**Points to be emphasized**	**Present√**	**Absent X**	**Comments**
Department Name			
Title of course/module/round			
Timing of Test (End of semester)			
Date of the exam			
Time allowed			
Total marks of the test			
Marks assigned to each item (question)<br>Marks assigned for subitems (if applicable)			
All questions to be answered			
Type of objective test items included:<br>– Single best answer<br>– True/False<br>– Extended matching<br>– Complete/Short answer			
Clear and comprehensive directions for each type of objective test items:<br>– Single best answer<br>– True/False<br>– Extended matching<br>– Complete/Short answer			
Total number of questions<br>Total number of exam papers<br>An instruction for students to check this before answering			

II. Evaluation of test items

MCQs

MCQ Content Validity:

**Cognitive domains (ILOs) to be assessed (Knowledge level)**	**Percentage of total**
Knowledge (Recall of isolated facts)	
Understanding (Comprehension)	
Application*	
Problem solving (Higher order thinking, reasoning)*	
**Total**	**100**

**Asking the question in the context of a clinical situation, diagram, graph, image, radiologic image, histopathological section, laboratory findings, et*

III. MCQ Item Construction:

	**Guidelines are followed**		**Guidelines are not followed.**	**Comments**
	**S** (Satisfactory)	**N** (Needs improvement	**U** (Unsatisfactory)	
**Stems**				
• Format used: *(tick all formats used)*<br>☐ Direct question<br>☐ Incomplete statement<br>☐ Problem, case or scenario<br>☐ Exhibits*/Visual representation (e.g., pictures, diagrams, graphs, radiologic images radiographs, tables, illustrations, etc.)<br>**Exhibits can present information in a way that's practical, clinically relevant, and concise. They also provide an alternative way to assess critical thinking.*<br>• The stem is written in a simple, clear and concise manner.				

• The stem is focused (can be answered without looking at the options).<br>• If the incomplete-sentence format is used, the options should flow directly and grammatically from the stem.				
**If a clinical scenario is used**,				
• only relevant information is included.<br>The stem includes a lead-in that states the task required of the candidate (e.g.: make a diagnosis, order a test, etc.).<br>• The problem to be solved should be clearly defined in the stem. Candidates should not have to look at the options to determine what the item is requesting.<br>• Avoid absolute terms (always, never, all, none).<br>• Avoid imprecise terms (seldom, rarely, sometimes, occasionally, few, many).<br>• Avoid cues (may, could, can).<br>• The stem is stated in positive form whenever possible (limited use of "not," "except").<br>• If negative wording is used in the stem, is it emphasized by capitalization, being bold and underlined.<br>• Avoid "tricky" and overly complex items.<br>• Define abbreviations, eponyms, or acronyms when used.				
**Guidelines for Correct or Best Answers**				
• The correct answer should be clearly the only correct one or better than the distractors.				

• If the *best* answer is sought, this should be clearly stated in the stem.<br>• Avoid clues to the correct answer:<br>• using textbook wording in the correct answer and not in the distractors<br>• using specific determiners such as *always*, *never*, etc.<br>• the correct answer contains the exact wording of an important concept mentioned in the stem<br>• there is no link between the stem and some of the options<br>• there is lack of parallelism among the options (grammatical, structural, vocabulary, technical jargon)				
**Options (Alternatives):**				
• The number of options is at least 4, and not more than 5.<br>• There is only one correct or best answer.<br>• Options are grammatically consistent with the stem.<br>• Options are homogenous (relate to each other e.g., all diagnoses, all tests, all treatments, etc.).<br>• Options are similar, as possible, to the correct answer in terms of grammar, length, and complexity.<br>• To eliminate length as a clue, the correct answer is about as long as one or more of the other options.<br>• The options are free from verbal clues to the correct answer.<br>• Options are plausible (logically consistent with the stem, with no silly or obviously incorrect ones).<br>• Use of "all of the above" and "none of the above" options is avoided or kept to a minimum (not more than 5%).<br>• No options combining two previous ones (e.g., A and C or B and D).				

• No verbal association between any of the options and the stem.<br>• The position of the correct answer is varied, so there’s no detectable pattern.<br>• Follow a logical order when appropriate (e.g., numbers, dates, ranges of values).<br>• Options are independent and do not overlap				

Technical Item Flaws

	**YES√**	**NO×**	**Comments**
**A. Issues Related to Testwiseness**			
• **Absolute terms** - terms such as “always” or “never” are in some options			
• **Grammatical cues** - one or more distractors don’t follow grammatically from the stem			
• **Logical cues** - a subset of the options is collectively exhaustive			
• **Long correct answer** - correct answer is longer, more specific, or more complete than other option			
• **Word repeats** - a word or phrase is included in the stem and in the correct answer			
• **Convergence strategy** - the correct answer includes the most elements in common with the other options			
B. **Issues Related to Irrelevant Difficulty**			
• Options are long, complicated, or double			

• Terms in the options are vague (eg, “rarely,” “usually”)			
• Language in the options is not parallel			
• Options are in a nonlogical order			
• “None of the above” is used as an option			
• Numeric data are not stated consistently			
• Stems are tricky or unnecessarily complicated			
• The answer to an item is “hinged” to the answer of a related item			
**C. General Guidelines for Item Construction**			
• Make sure the item can be answered without looking at the options OR that the options are 100% true or false.			
Include as much of the item as possible in the stem; the stems should be long and the options short.			
And most important of all: Focus on important concepts; don’t waste time testing trivial facts.			
Avoid “tricky” and overly complex items			
Write options that are grammatically consistent and logically compatible with the stem; list them in logical or alphabetical order			
Write distractors that are plausible and the same relative length as the answer.			
Avoid using absolutes such as *always*, *never*, and *all* in the			

options; also avoid using vague terms such as *usually* and *frequently*.			
• Avoid negatively phrased items (eg, those with *except* or *not* in the lead-in). If you must use a negative stem, use only short (preferably single word) options.			

EMQ checklist

	**Guidelines are followed**		**Guidelines are not followed**
	**S (1)**<br><br>**(Satisfactory)**	**N (2)**<br><br>**(Needs improvement)**	**U (3)**<br><br>**(Unsatisfactory)**
**A. Theme**			
**1. Theme is a general topic or title (body system – chief complaint – class of drugs – anatomic sites …)**			
**2. Theme is a short title (Fatigue – Headache….)**			
**B. Option list**			
**3. Option list ranges from 6 – 25**			
**4. All options are relevant and appropriate level of difficulty**			
**5. Options are short and focused list (normally only one or two words)**			
**6. Options are listed in logical / alphabetical order**			

7. Options are homogenous (all treatments, diagnoses…)			
8. Avoid esoteric options. (Zebras)			
9. Avoid overlapping options.			
10. Only one best answer for each item stem, with several plausible distractors.			
**C. Lead in statement**			
11. Lead in statement is clear and focused and indicates the relationship between the stem and options			
12. A single lead in statement is used for all stems			
13. Avoid nonspecific lead in statements: (Match each item with the correct options)			
14. Lead in statement specifies how to select the options. (Each option can be used once, more than once or not at all.)			
**D. Item stems**			
15. Two or more item stems are included.			
16. Clear and Focused vignette were used.			
17. Avoid non vignette items as it focuses on recall of isolated facts.			
18. Stems are similar in structure to minimize cuing.			

**19. Stems could be answered without looking to option list. (Cover test) indicating well written stems.**			
